# The structure and properties of PEDOT synthesized by template-free solution method

**DOI:** 10.1186/1556-276X-9-557

**Published:** 2014-10-07

**Authors:** Qin Zhao, Ruxangul Jamal, Li Zhang, Minchao Wang, Tursun Abdiryim

**Affiliations:** 1 Key Laboratory of Petroleum and Gas Fine Chemicals, Educational Ministry of China, School of Chemistry and Chemical Engineering, Xinjiang University, Urumqi 830046, People's Republic of China; 2 Key Laboratory of Functional Polymers, Xinjiang University, Urumqi 830046, People's Republic of China; 3 Key Laboratory of Oil and Gas Fine Chemicals, Educational Ministry of China, College of Chemistry and Chemical Engineering, Xinjiang University, Urumqi 830046, People's Republic of China

**Keywords:** Template-free method, PEDOT, Different morphology, Structure and properties

## Abstract

In this study, a simple one-step template-free solution method was developed for the preparation of poly(3,4-ethylenedioxythiophene) (PEDOTs) with different morphologies by adjusting various ratios of oxidant (FeCl_3_·6H_2_O) to monomer (3,4-ethylenedioxythiophene (EDOT)). The results from structural analysis showed that the structure of PEDOT was strongly affected by the oxidant/monomer ratio, and the polymerization degree, conjugation length, doping level, and crystallinity of PEDOT decreased with increasing of the oxidant/monomer ratio. The morphological analysis showed that PEDOT prepared from an oxidant/monomer ratio of 3:1 displayed a special coral-like morphology, and the branches of ‘coral’ would adjoin or grow together with increasing content of oxidant in the reaction medium; consequently, the morphology of PEDOT changed from coral to sheets (at an oxidant/monomer ratio of 9:1). The electrochemical analysis proved that the PEDOT prepared from an oxidant/monomer ratio of 3:1 had the lowest resistance and the highest specific capacitances (174 F/g) at a current density of 1 A/g with a capacity retention rate of 74% over 1,500 cycles, which indicated that the PEDOT with a coral-like morphology could be applied as a promising electrode material for supercapacitors.

## Background

With the globalization of economy, the increasing demand for portable systems, miniaturized wireless sensor networks, electric vehicles, and hybrid electric vehicles has spawned great interest in electrochemical capacitors, also known as supercapacitors [1-3]. Electrochemical supercapacitors with relatively high energy and power densities are part of the significant field of electrochemical energy storage because they bridge the gap between conventional capacitors and batteries. Besides, supercapacitors have a long lifespan, rapid charge/discharge rate, and wide operating temperature range [4-8]. Typically, a supercapacitor includes electrodes, separators, and plastic outer package [9,10]. The electrodes are the key to a supercapacitor, which are generally made from carbon materials, transition metal oxides, and conducting polymers. In general, the conducting polymers have an advantage over carbon materials or transition metal oxides due to their good environmental stability, high conductivity, high transparency, and low oxidation potential [11].

Among conducting polymers, poly(3,4-ethylenedioxythiophene) (PEDOT) exhibits both an unusual stability and a high conductivity at an oxidized state, which has been considered as perhaps the most stable conducting polymer currently available [12]. So far, many researchers have studied to further improve the capacitive performances of supercapacitors by increasing the specific surface area of electrode materials. As known, PEDOT exhibits an irregular amorphous shape by most methods [13,14]. Most of the methods to prepare special nanostructures of PEDOT include template synthesis, reverse microemulsion polymerization, and interfacial polymerization in the presence of a surfactant. For example, Hu et al. synthesized a 3D flowerlike PEDOT which shows a specific capacitance of 111 F/g by using bis(2-ethylhexyl) sulfosuccinate sodium as surfactant [15]. Lacroix et al. reported the electrodeposition of PEDOT films from an aqueous surfactant solution through a two-dimensional poly(styrene) template onto an indium tin oxide substrate [16]. Han et al. synthesized conducting PEDOT:PSS nanospheres onto a flexible poly(ethylene terephthalate) substrate [17]. However, it is difficult to obtain a PEDOT nanostructure with high conductivity by a simple chemical solution method without a template. Nabid et al. synthesized PEDOTs with fiber- and sphere-like morphologies by template-free route in the presence of different oxidants; however, all PEDOTs exhibited a low crystallinity and oxidation degree (or doping level), resulting in a decrease in conductivity of PEDOT [18].

In this paper, we report a simple one-step template-free solution method for the preparation of PEDOTs with different morphologies by adjusting various ratios of oxidant (FeCl_3_·6H_2_O) to monomer (3,4-ethylenedioxythiophene (EDOT)). The polymerization was carried out in a template-free alcoholic aqueous solution medium. The correlation between the structures and properties of the PEDOTs prepared from different contents of oxidant was discussed based on the results from Fourier transform infrared (FTIR) spectroscopy, ultraviolet-visible (UV-vis) spectroscopy, Raman spectroscopy, X-ray diffraction (XRD), field-emission scanning electron microscopy (FESEM), and transmission electron microscopy (TEM). The possible mechanism for the formation of different morphologies of PEDOT from various ratios of oxidant to EDOT was proposed. Moreover, the galvanostatic charge/discharge measurement, cyclic voltammetry (CV), electrochemical impedance spectroscopy (EIS), and cycle life measurement were done to evaluate the potential application of PEDOTs as a supercapacitor material.

## Methods

### Materials

EDOT was obtained from Shanghai Aladdin Reagent Company (Shanghai, China) and stored in a refrigerator prior to use. FeCl_3_·6H_2_O was obtained from Tianjin Zhiyuan Chemical Reagent Co., Ltd (Tianjin, China). All other reagents were of analytical grade and used as supplied without further purification.

### Preparation of PEDOT

A typical synthesis process of PEDOT was carried out in the following steps: 20 mL FeCl_3_·6H_2_O (1.0 M) aqueous solution was added in a 50-mL beaker with a magnetic stirrer. Then, 5.0 mL of 14.2 wt.% EDOT alcoholic solution was quickly added into the above solution. There was an obvious phenomenon that the orange solution of FeCl_3_·6H_2_O became black after adding the EDOT alcoholic solution. The mixing solution was under vigorous stirring for 24 h, then filtered and washed by absolute ethyl alcohol and distilled water, and at last dried at 60°C for 24 h. The obtained polymer was noted as PEDOT (3:1). In a similar manner, the molar ratio of oxidant to monomer (represented by [FeCl_3_·6H_2_O]/[EDOT]) was adjusted at 6:1 and 9:1, respectively; the resultant polymers were noted as PEDOT (6:1) and PEDOT (9:1).

### Apparatus

The FTIR spectra of the polymers were obtained using a Bruker Equinox 55 Fourier transform infrared spectrometer (Bruker, Billerica, MA, USA) (frequency range 4,000 to 500 cm^-1^). The UV-vis spectra of the samples were recorded on a UV-vis spectrophotometer (UV4802, Unico, Dayton, NJ, USA). Raman spectra were recorded in a backscattering geometry with a 1,064-nm excitation wavelength using a Bruker Vertex 70 FT infrared spectrometer (equipped with RamIIFT Raman Module). XRD patterns have been obtained using a Bruker AXS D8 diffractometer with a monochromatic Cu-Kα radiation source (*λ* = 0.15418 nm); the scan range (2*θ*) was 10° to 80°. Morphology and microstructure of the samples were investigated by FESEM (Hitachi S-4800, Hitachi Ltd., Chiyoda-ku, Japan). TEM measurements were performed on a TEM instrument (JEOL model 2100, JEOL Ltd., Tokyo, Japan).

### Electrochemical test

The electrodes were prepared by mixing 85 wt.% active materials (3 mg), 10 wt.% carbon black, and 5 wt.% polytetrafluoroethylene (PTFE) to form a slurry. The slurry was pressed on a graphite current collector (area, 1 cm^2^) and then dried at 60°C for 24 h. All electrochemical experiments were carried out using a three-electrode system, in which the sample was used as the working electrode, platinum as the counter electrode, and saturated calomel electrode (SCE) as the reference electrode, and 1 M H_2_SO_4_ was used as the electrolyte. The CV, galvanostatic charge/discharge tests were done in the potential window ranging from -0.2 to 0.8 V by using CHI 660C electrochemical workstation (CH Instruments Inc., Shanghai, China). EIS measurements were performed by using CHI 660C electrochemical workstation in the frequency range of 0.01 Hz to 100 kHz. The cycle life measurement of polymers was recorded by sequential CV cycling (over 1,500 cycles) at a scan rate of 10 mV/s.

## Results and discussion

**Figure 1:**
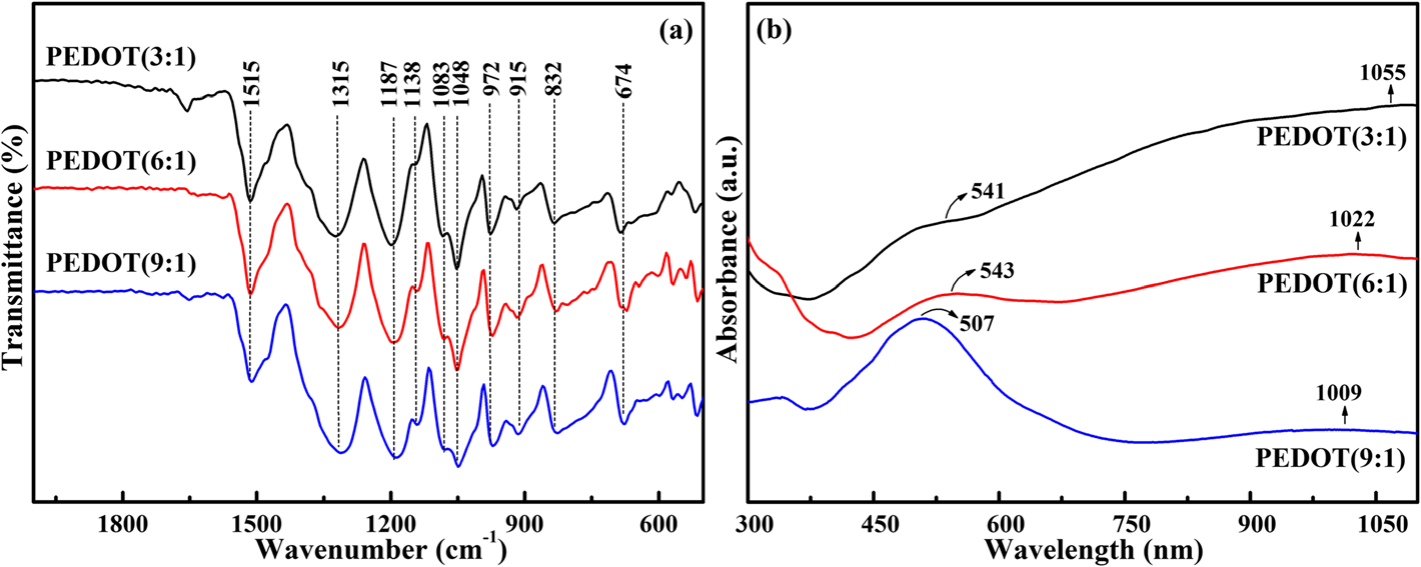
FTIR spectra (a) and UV-vis spectra (b) of PEDOT (3:1), PEDOT (6:1), and PEDOT (9:1).

Figure 1a shows the FTIR spectra of PEDOTs. All the spectra show the typical bands for PEDOT. The mentioned bands are as follows: 1,515, 1,315, 1,187, 1,138, 1,083, 1,048, 972, 915, 832, and 674 cm^-1^. The bands at approximately 1,515 and 1,315 cm^-1^ are assigned to asymmetric stretching mode of C = C and inter-ring stretching mode of C-C, respectively. The bands appearing at around 1,187, 1,138, 1,083, and 1,048 cm^-1^ are attributed to the C-O-C bending vibration in ethylenedioxy group, while the bands at 972, 915, 832, and 674 cm^-1^ are the characteristic bands of stretching vibrations of the C-S-C bond in thiophene ring, which suggests the successful formation of the PEDOT in this polymerization reaction [19-21]. According to the previous report, the degree of polymerization of polythiophene can be evaluated from the ratio of integration of the infrared bands at 670 and 830 cm^-1^, and the higher degree of polymerization resulted from a relatively lower value of that intensity ratio [22,23]. It can be deduced from Figure 1a that the intensity ratio of integration of the infrared bands at 670 and 830 cm^-1^ are in the order of PEDOT (3:1) < PEDOT (6:1) < PEDOT (9:1), indicating that PEDOT (3:1) has the highest polymerization degree.

Figure 1b shows the UV-vis absorption spectra of PEDOTs. As seen from Figure 1b, the different molar ratios of oxidant to monomer bring different absorption spectra. All PEDOTs show two characteristic peaks at approximately 400 to 700 nm and approximately 1,000 to 1,060 nm with a free tail extending into the near-infrared region. The peaks at approximately 400 to 700 can be ascribed to the π-π* transitions of thiophene ring [24], whereas the peaks at approximately 1,000 to 1,060 nm can be attributed to polaron and/or bipolaron bands, which are characteristic of oxidized PEDOT with high conjugation length [25-28]. On comparison, decreasing of the [FeCl_3_·6H_2_O]/[EDOT] ratio causes a redshift in the π-π* absorption peak from 507 to 541 nm. This implies that the oxidation degree and conjugation length of PEDOT increase with decreasing of the [FeCl_3_·6H_2_O]/[EDOT] ratio. Moreover, the intensity ratio of the polaron and/or bipolaron band to the π-π* transition band (*I*_1,000-1,060_/*I*_400-700_) of the PEDOT (3:1) is higher than that of others, suggesting that the highest doping level occurs in the case of PEDOT (3:1) [25-28].

**Figure 2:**
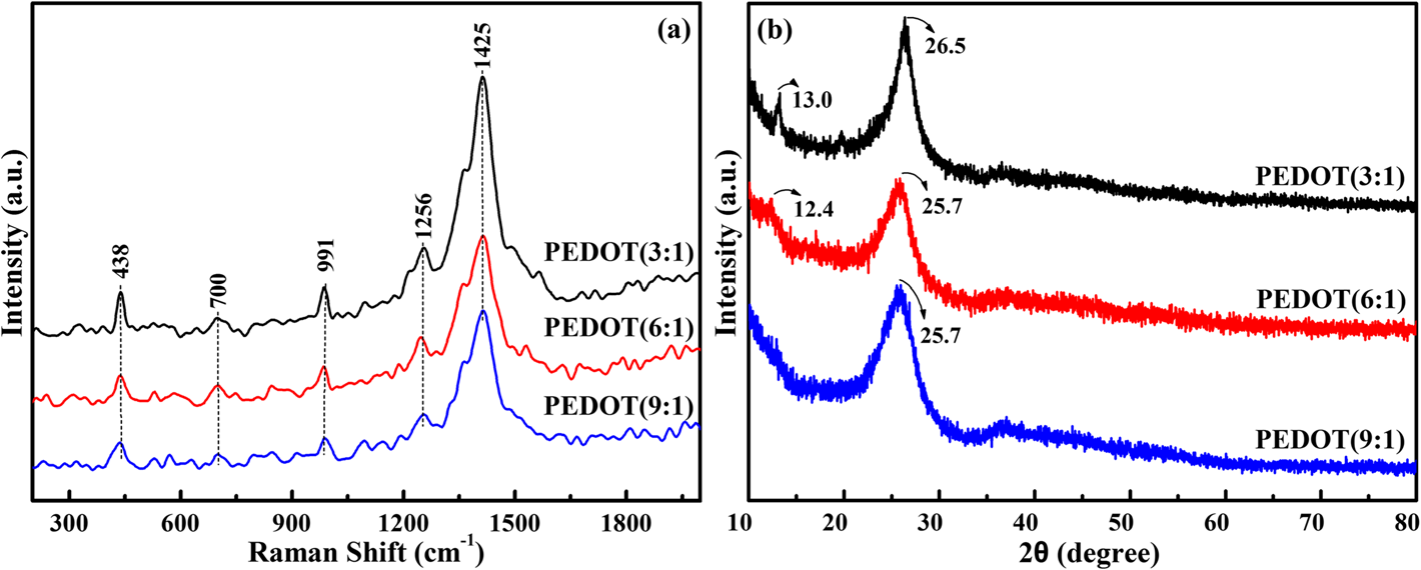
Raman spectra (a) and XRD patterns (b) of PEDOT (3:1), PEDOT (6:1), and PEDOT (9:1).

Some information about the structure of PEDOTs has been achieved by the analysis of Raman spectra (*λ*_exc_ =1,064 nm). As seen from Figure 2a, the bands at 438, 700, 991, and 1,256 cm^-1^ are assigned to the C-O-C deformation, symmetric C-S-C deformation, oxyethylene ring deformation, and C_α_-C_α_ inter-ring stretching, respectively. Especially, the characteristic band at 1,425 cm^-1^ due to symmetric C_α_ = C_β_ (-O) stretching is indicative of a high level of conjugation in the structure of PEDOT [29-31]. Lee et al. have already found that a characteristic band corresponding to the stretching vibration of C = C becomes narrow, which could prove a change of resonance structure of the PEDOT chain from a benzoid to a quinoid [32]. A similar phenomenon is observed for PEDOT (3:1), in which the band at 1,425 cm^-1^ becomes narrow, implying that PEDOT (3:1) has a longer degree of conjugate length.

**Figure 3:**
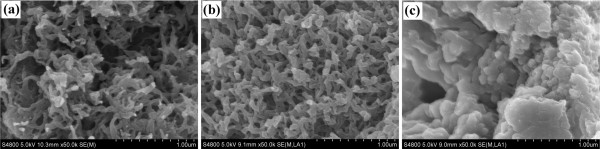
FESEM images of (a) PEDOT (3:1), (b) PEDOT (6:1), and (c) PEDOT (9:1).

**Figure 4:**
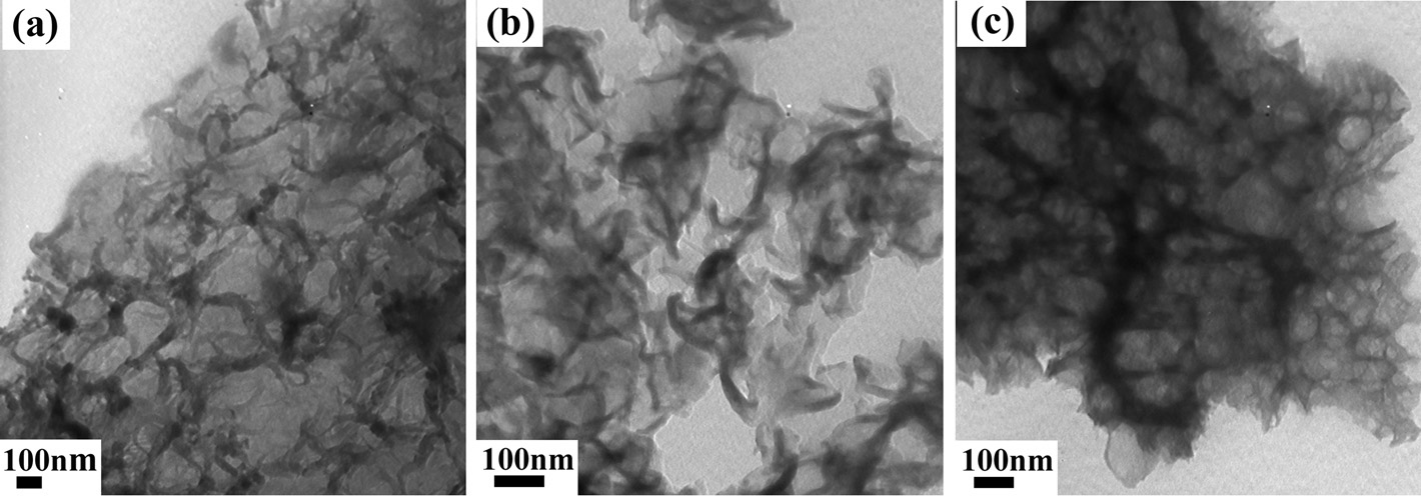
TEM images of (a) PEDOT (3:1), (b) PEDOT (6:1), and (c) PEDOT (9:1).

XRD patterns of PEDOTs are shown in Figure 2b. All the polymers show characteristic peak at nearly 2*θ* ~ 26°, which can be attributed to the interchain planar ring stacking [33,34]. Furthermore, the diffraction peaks with low intensity at 2*θ* ~ 13° and 2*θ* ~ 12.4° occur in the case of PEDOT (3:1) and PEDOT (6:1). Generally, the peak at 2*θ* ~ 13° is considered to be the distance between two stacks in the two-dimensional stacking arrangement of polymer chains and intervening dopant ions [35]. A comparison indicates that the diffraction peak of PEDOT (3:1) at 2*θ* ~ 26.5° becomes sharper and more intense, revealing that PEDOT (3:1) has a higher crystallinity than other PEDOTs.Figures 3 and 4 show the SEM and TEM images of PEDOTs, respectively. As depicted in Figures 3a and 4a, PEDOT (3:1) displays a coral-like morphology with many tentacles which are separated. Figures 3b and 4b show PEDOT (6:1) with a more intensive coral-like morphology, and the tentacles adhere with each other in some part. Moreover, PEDOT (9:1) has a flake-like morphology in Figures 3c and 4c. It can be concluded that the morphology of PEDOT displays a regular variation with increasing concentration of oxidant.

**Figure 5:**
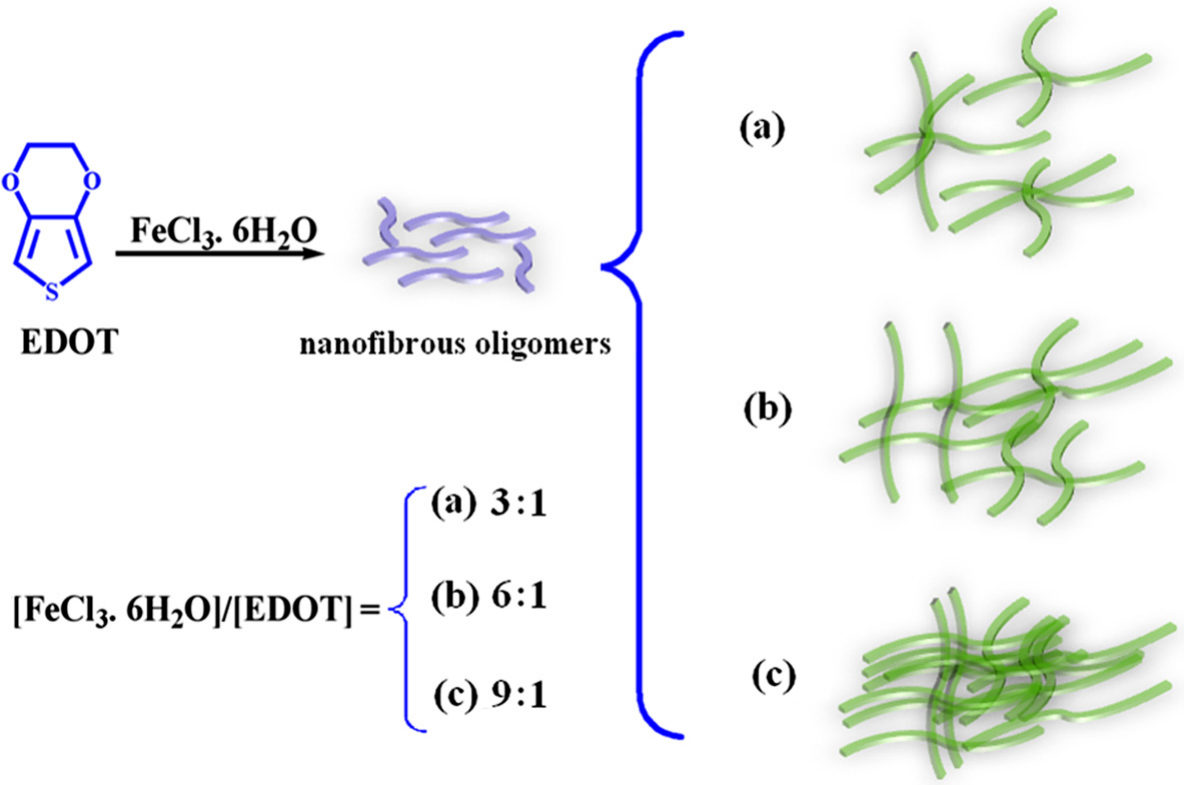
**The possible mechanism for formation of the different morphologies of PEDOT. (a)** 3:1, **(b)** 6:1, and **(c)** 9:1.

Figure 5 shows the possible mechanism for formation of different morphologies of PEDOT to explain the regular variation at various ratios of oxidant (FeCl_3_·6H_2_O) to the EDOT. According to the previous report, the quick propagation of PEDOT chains happens in a limited time under rapid initiated condition, and a lot of initial nanofibrous oligomers can be formed in the early stage [18]. Furthermore, as the polymerization time increases, the EDOT species including the monomer and the newly formed nanofibrous oligomers can act as a soft template for the formation of the nanofibrous structures [18]. Therefore, it can be concluded that the coral-like morphology of PEDOT occurring in the case of molar ratio of [FeCl_3_·6H_2_O]/[EDOT] at 3:1 mainly resulted from nanofibers growing together (or twisted nanofibers), which are formed from a soft template effect of nanofibrous oligomers. When increasing the concentration of FeCl_3_·6H_2_O, the content of monomer and the newly formed nanofibrous oligomers is relatively reduced. And with increasing content of FeCl_3_·6H_2_O, the branches of ‘coral’ will adjoin or grow together and consequently lead to a morphology of mixing up tentacles with sheets in the case of the molar ratio of [FeCl_3_·6H_2_O]/[EDOT] at 6:1, a and sheet-like morphology for PEDOT from the molar ratio of [FeCl_3_·6H_2_O]/[EDOT] at 9:1.

**Figure 6:**
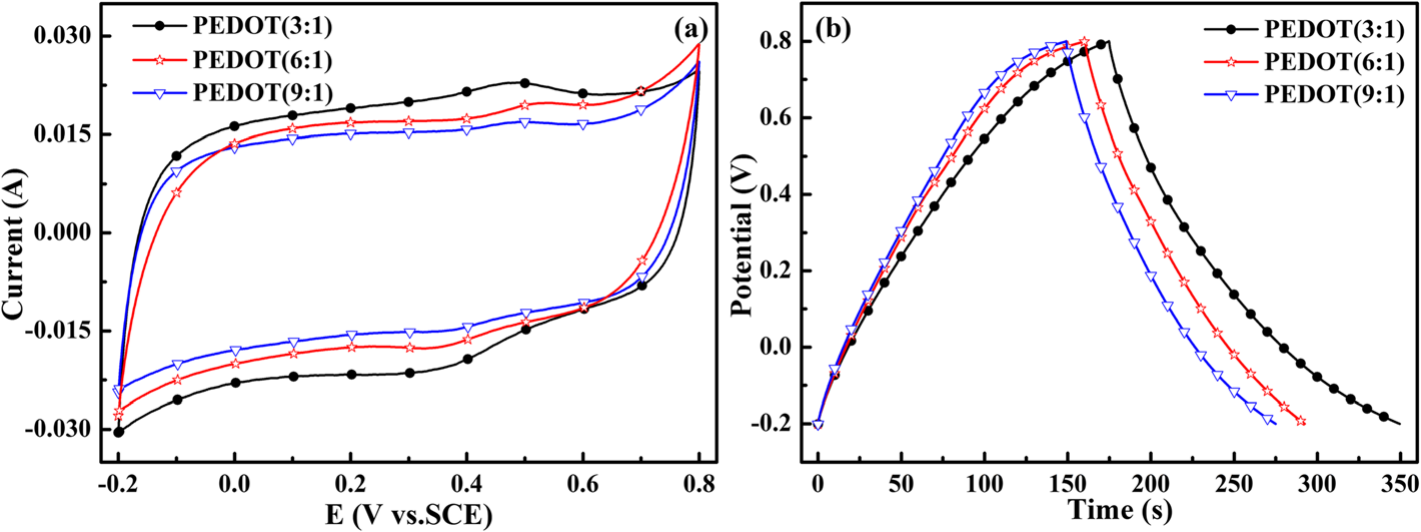
**Electrochemical performance of PEDOT is performed in 1 mol L**^**-1 **^**H**_**2**_**SO**_**4**_**.** Mass of the active material =3 mg; **(a)** CV curves of PEDOT (3:1), PEDOT (6:1), and PEDOT (9:1) at a scan rate of 50 mV s^-1^. **(b)** Galvanostatic charge/discharge curves of PEDOT (3:1), PEDOT (6:1), and PEDOT (9:1) at a current density of 3 mA cm^-2^.

The dependence of specific capacitance with potential of the PEDOT powder at a scan rate of 50 mV/s is shown in Figure 6a. All the scans are performed in a potential range from -0.2 to 0.8 V. As depicted in Figure 6a, one pair of redox can be observed for PEDOTs. The relevant anodic and cathodic peaks of PEDOT (3:1), PEDOT (6:1), and PEDOT (9:1) are (0.46 V, 0.33 V), (0.53 V, 0.34 V), and (0.49 V, 0.35 V), respectively. Compared with PEDOT (6:1) and PEDOT (9:1), it is clear to see the positive shift in the reduction peak potential and the negative shift in the oxidation peak potential of the PEDOT (3:1). Generally, these shifts in anodic and cathodic peak potential can be explained by the higher oxidation degree of PEDOT (3:1) than PEDOT (6:1) and PEDOT (9:1), and the higher oxidation degree leads to an increase in the conductivity of polymer chains, which in turn brings a decrease in the oxidation resistance of the polymer chains. Generally, a method to evaluate the resistance of the electrode material depends on the shape of the capacitance loop of the supercapacitor. The shape of the capacitance loop is almost rectangular, and the electrode material has a lower resistance. On the other hand, the electrode material with high resistance distorts the rectangular shape to become an oblique angle [36]. It is worthy to note that the capacitance loop of PEDOT (3:1) is very close to being rectangular, indicating an excellent capacitance behavior and low internal resistance in the electrode. Moreover, it is observed from Figure 6a that PEDOT (3:1) has the largest rectangular area among the PEDOTs, suggesting that PEDOT (3:1) has better electrochemical behavior and higher capacitance than others.

Figure 6b shows the galvanostatic charge/discharge curves of PEDOTs performed at a current density of 3 mA cm^-2^ in the three-electrode system between -0.2 and 0.8 V; the supporting electrolyte is 1 M H_2_SO_4_. The specific capacitance (SC) of the electrode materials is calculated by means of SC = (*I* × Δ*t*)/(Δ*V* × *m*) [37], where *I* is the charge/discharge current, Δ*t* is the discharge time, Δ*V* is the electrochemical window (1 V), and *m* is the mass of active materials within the electrode (3 mg). The SC of PEDOT (3:1), PEDOT (6:1), and PEDOT (9:1) is 174, 132, and 124 F/g, respectively. This result is consistent with the CV analysis. It should be noted that the SC of PEDOT (3:1) is higher when compared with other reports [38-40]. Furthermore, PEDOT (3:1) also has both longer charge and discharge time and has a high charge/discharge efficiency (*η*) of 98.8%, while the charge/discharge efficiency of PEDOT (6:1) and PEDOT (9:1) is 81.9% and 83.0%, respectively. The results confirm that PEDOT (3:1) has a better capacitance behavior [4]. This enhanced SC of the PEDOT (3:1) electrode can be attributed to the fact that the coral-like morphology of the polymer offers a higher specific surface area which is convenient for ions accessing into the polymer matrix and inducing higher charge to keep stable. And, the special coral structure provides broader room and shorter diffusion distances for ion transport between the electrolyte and PEDOT molecules.

**Figure 7:**
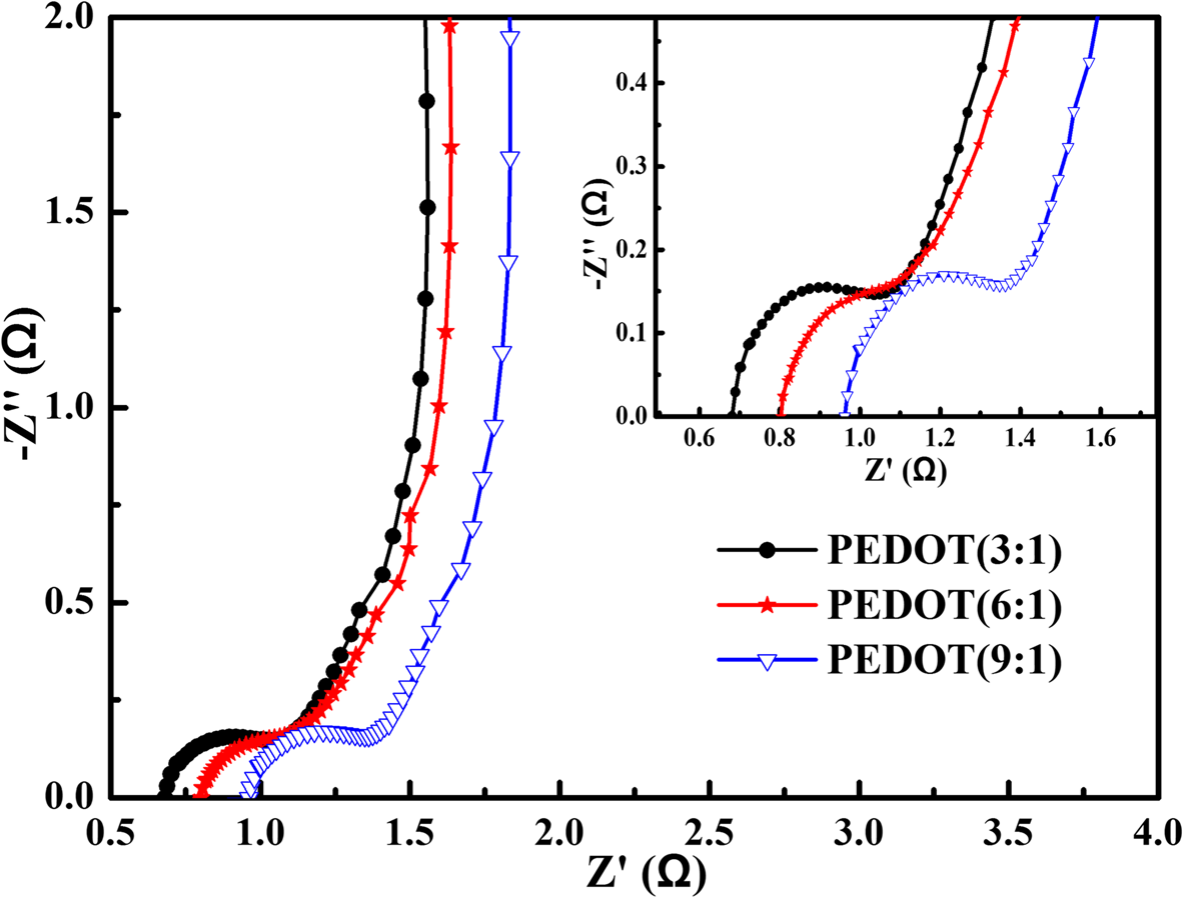
**EIS spectra of PEDOT (3:1), PEDOT (6:1), and PEDOT (9:1) at 5 mV.** Frequency range =0.01 Hz to 100 KHz.

Nyquist plots of PEDOTs at 5 mV over the frequency range of 0.01 Hz to 100 KHz are given in Figure 7. It can be seen that EIS plots contain three well-separated patterns. Firstly, the high-frequency intercept of the semicircle with the real axis can be used to evaluate the value of internal resistance, which included the resistance of the electrolyte solution, the intrinsic resistance of the active material, and the contact resistance at the interface active material/current collector [41,42]. The values of solution resistance (*R*_s_) obtained from Figure 7 are 0.68 Ω (PEDOT (3:1)), 0.80 Ω (PEDOT (6:1)), and 0.96 Ω (PEDOT (9:1)), respectively. It is clear that the internal resistance of PEDOT (3:1) is relatively lower among PEDOTs. The radius of the semicircle represents the charge transfer resistance (*R*_ct_) as can be seen from Figure 7. The charge transfer resistance (*R*_ct_) is 0.14 Ω (PEDOT (3:1)), 0.15 Ω (PEDOT (6:1)), and 0.16 Ω (PEDOT (9:1)), respectively. The small semicircle might be due to the diffusion effect of the electrolyte in the electrodes. Secondly, in the middle-frequency regime, an extremely small 45° inclination can be seen (Warburg response) which arises as a result of distributed capacitance/impedance in a porous material. Thirdly, at low frequencies, the vertical line indicates the pure capacitive behavior (*R*_p_); the more vertical curve suggests the better capacitive behavior of the supercapacitor [43]. It can be seen that the inclined line with a slope is closer to 90° for the PEDOT (3:1), which was a characteristic feature of pure capacitive behavior [44]. All the results also further illustrate that PEDOT (3:1) shows lower resistances and a better capacitive behavior.

**Figure 8:**
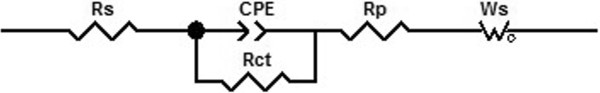
**Equivalent circuit diagram to fit the observed impedance spectra in Figure**7**.**

**Figure 9:**
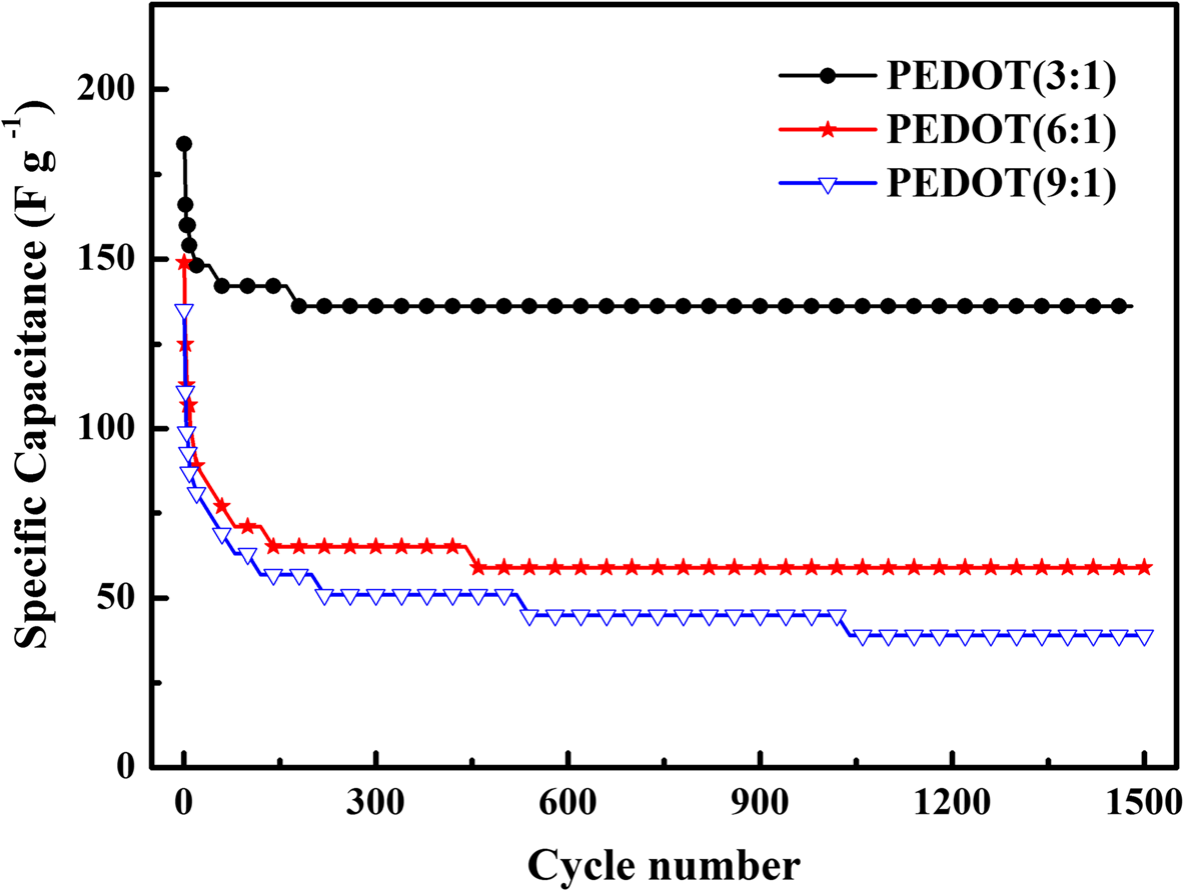
Cycle stability curves of PEDOT (3:1), PEDOT (6:1), and PEDOT (9:1).

The equivalent circuit diagram used to fit the impedance spectrum is shown in Figure 8. All the impedance curves obtained are basically fitted with this unique equivalent circuit. It is composed of two frequency parts in Figure 7. A resistance of *R*_s_ (solution resistance) is in series with a parallel element circuit constant phase element (CPE) and a resistor *R*_ct_ (Faradic charge transfer resistance), which can be attributed to the sum of electrolytic and the electrode material for the high-frequency semi-cycle observed in Figure 7. The second part element circuit of a resistor *R*_p_ (capacitor resistance) and the *W*_s_ (ionic diffusion Warburg impedance) can be attributed to the low-frequency domain.As it is well known, the long-term stability of conducting polymers is an important factor to consider for their applications in supercapacitors. The cycling performance of PEDOT with different structures is evaluated by repeating the galvanostatic charge/discharge test at the 1 A/g for 1,500 cycles in Figure 9. Among samples, PEDOT (3:1) displayed excellent capacity retention with only 26% decrease over 1,500 cycles. In addition, at the first 10 cycles, the SC of PEDOT (3:1) decreases sharply from 184 to 154 F/g, and it decreases to 74% after 180 cycles. However, PEDOT (6:1) and PEDOT (9:1) exhibit decay rapidly from 149 to 59 F/g and 135 to 40 F/g, respectively. Besides, PEDOT (3:1) reaches a stability state after 200 cycles, but the specific capacitance of PEDOT (6:1) and PEDOT (9:1) reaches the stability state after 460 and 1,040 cycles, respectively.

All electrochemical testing suggests that PEDOT (3:1) has the promising electrocapacitive property among PEDOTs. This enhanced SC of the PEDOT (3:1) electrode can be attributed to the following reasons: (1) the highest polymerization degree, doping level, conjugation length, and crystallinity occurring in the case of PEDOT (3:1) among PEDOTs, which can enhance the conductivity of polymer chains, and (2) the coral-like morphology of PEDOT (3:1), which offers a high specific surface area for convenient ions accessing into the polymer matrix and inducing higher charge to keep stable. And, the special coral structure provides broader room and shorter diffusion distances for ion transport between the electrolyte and PEDOT molecules.

## Conclusions

In this study, a simple one-step template-free solution method was developed for the preparation of PEDOTs with different morphologies by adjusting various ratios of oxidant (FeCl_3_·6H_2_O) to monomer (EDOT). The polymerization was carried out in a template-free alcoholic aqueous solution medium. The results showed that the alcoholic aqueous solution medium was beneficial for the dispersion of EDOT and the uniform contact between EDOT and FeCl_3_·6H_2_O, and this would increase the possibility of formation of PEDOT with high polymerization degree, conjugation length, doping level, and crystallinity. The results also showed that the coral-like morphology of PEDOT occurring in the case of the molar ratio of [FeCl_3_·6H_2_O]/[EDOT] at 3:1 mainly resulted from nanofibers growing together. And, with increasing content of FeCl_3_·6H_2_O, the branches of ‘coral’ would adjoin or grow together and consequently lead to a morphology of mixing up tentacles with sheets ([FeCl_3_·6H_2_O]/[EDOT] at 6:1) and a sheet-like morphology ([FeCl_3_·6H_2_O]/[EDOT] at 9:1). With the highest polymerization degree, conjugation length, and crystallinity, PEDOT prepared from [FeCl_3_·6H_2_O]/[EDOT] at 3:1 displayed a higher conductivity, a lower internal resistance, and a stable cycling performance. This enhanced behavior which could be attributed to the special coral structure will provide broader room and shorter diffusion distances for ion transport between the electrolyte and PEDOT molecules, which demonstrated that PEDOT with coral-like morphology displayed an excellent electrochemical capacitive behavior and could be used as an electrode material for supercapacitors.

## Competing interests

The authors declare that they have no competing interests.

## Authors' contributions

QZ conceived the study, carried out the data analysis, and drafted the manuscript. RJ carried out the sample preparation and the experimental measure. LZ and MW participated in the study of material structures and the data analysis. TA coordinated the research and revised and finalized the manuscript. All authors read and approved the final version of the manuscript.
